# How to sensitize glioblastomas to temozolomide chemotherapy: a gap-centered view

**DOI:** 10.3389/fcell.2024.1436563

**Published:** 2024-07-01

**Authors:** Alila Miramova, Anton Gartner, Dmitri Ivanov

**Affiliations:** ^1^ Department of Biological Sciences, Ulsan National Institute of Science and Technology (UNIST), Ulsan, Republic of Korea; ^2^ Graduate School for Health Sciences and Technology, Ulsan National Institute of Science and Technology (UNIST), Ulsan, Republic of Korea; ^3^ Center for Genomic Integrity, Institute for Basic Science, Ulsan, Republic of Korea

**Keywords:** temozolomide, chemotherapy, glioblastoma, PARP inhibitor, ssDNA gaps, mismatch repair

## Abstract

Temozolomide (TMZ) is a methylating agent used as the first-line drug in the chemotherapy of glioblastomas. However, cancer cells eventually acquire resistance, necessitating the development of TMZ-potentiating therapy agents. TMZ induces several DNA base adducts, including *O* ^*6*^-meG, 3-meA, and 7-meG. TMZ cytotoxicity stems from the ability of these adducts to directly (3-meA) or indirectly (*O* ^*6*^-meG) impair DNA replication. Although TMZ toxicity is generally attributed to *O* ^*6*^-meG, other alkylated bases can be similarly important depending on the status of various DNA repair pathways of the treated cells. In this mini-review we emphasize the necessity to distinguish TMZ-sensitive glioblastomas, which do not express methylguanine-DNA methyltransferase (MGMT) and are killed by the futile cycle of mismatch repair (MMR) of the *O* ^*6*^-meG/T pairs, vs. TMZ-resistant MGMT-positive or MMR-negative glioblastomas, which are selected in the course of the treatment and are killed only at higher TMZ doses by the replication-blocking 3-meA. These two types of cells can be TMZ-sensitized by inhibiting different DNA repair pathways. However, in both cases, the toxic intermediates appear to be ssDNA gaps, a vulnerability also seen in BRCA-deficient cancers. PARP inhibitors (PARPi), which were initially developed to treat BRCA1/2-deficient cancers by synthetic lethality, were re-purposed in clinical trials to potentiate the effects of TMZ. We discuss how the recent advances in our understanding of the genetic determinants of TMZ toxicity might lead to new approaches for the treatment of glioblastomas by inhibiting PARP1 and other enzymes involved in the repair of alkylation damage (e.g., APE1).

## Temozolomide and its cytotoxicity

Temozolomide (TMZ) is a chemotherapeutic drug well suited for the therapy of glioblastomas since it readily penetrates the blood-brain barrier (Kaina and Christmann, 2019). Therapeutically achievable plasma TMZ concentrations of 30–100 μM (Ostermann et al., 2004; Gupta et al., 2014) are sufficient to eliminate TMZ-sensitive cancers but are well tolerated by normal cells. TMZ belongs to the group of S_N_1 (first-order nucleophilic substitution) methylating agents, which include *N*-methyl-*N*-nitrosourea (MNU) and *N*-methyl-*N’*-nitro-*N*-nitrosoguanidine (MNNG). TMZ hydrolysis produces 5-(3-methyl 1-triazenyl) imidazole-4-carboxamide (MTIC), which has a half-life of only 2 min and releases a highly reactive methyldiazonium ion, which methylates nucleophilic N or O atoms of the DNA bases. The most toxic TMZ-induced DNA base adduct is *O* ^*6*^-meG. Although TMZ induces several other base adducts, these are of minor importance in the killing of primary TMZ-sensitive tumors due to the high toxicity of *O* ^*6*^-meG. *O* ^*6*^-meG is capable of pairing not only with cytosine but also with thymine. *O* ^*6*^-meG adducts mispaired with thymine during the first round of DNA replication after TMZ treatment are recognized by the mismatch repair (MMR) pathway. MMR corrects mismatched bases only in the newly synthesized strands. Thus, MMR will excise thymine while leaving the initial lesion, i.e., *O* ^*6*^-meG, intact. Since *O* ^*6*^-meG is left unrepaired, it continues to pair with thymine during gap filling. Repetitions of this process are referred to as “futile cycles” of MMR. The resultant persistent ssDNA gaps interfere with the second round of DNA replication (Stojic et al., 2004). Although DNA damage signaling leading to apoptosis induction was also proposed as a possible explanation for the MMR-induced cell death (Yoshioka et al., 2006), according to the “classical” model, replication fork progression across unrepaired nicks results in dsDNA breaks, which are highly toxic to the cell. Remarkably, the same model was initially proposed for the mechanism of cell killing by PARP inhibitors, which are currently tested in clinical trials as enhancers of TMZ (Table 1).

**TABLE 1:** Ongoing clinical trials testing PARP inhibitors in glioblastoma chemotherapy.

Name of PARP inhibitor	NCT ID	Name of study	Location	Phase	Enrollment	Status	Completion
AZD9574	NCT05417594	A modular phase I/IIa, open-label, multi-centre study to assess the safety, tolerability, pharmacokinetics, pharmacodynamics and preliminary efficacy of ascending doses of AZD9574 as monotherapy and in combination with anti-cancer agents in patients with advanced solid malignancies (CERTIS1)	United States, Australia, Germany, Republic of Korea, Spain, Sweden, United Kingdom	1, 2	490	R	January 2026
Niraparib	NCT05076513	A phase 0 “trigger” trial of niraparib in newly-diagnosed glioblastoma and recurrent IDH1/2 (+) ATRX mutant glioma	United States	1	42	R	February 2025
Niraparib	NCT04221503	A phase II study evaluating the efficacy and safety of niraparib and tumor-treating fields in recurrent glioblastoma	United States	2	30	ANR	December 2025
Niraparib	NCT06258018	A phase I-II study of niraparib plus temozolomide “1 week on, 1 week off” in patients with recurrent Isocitrate Dehydrogenase (IDH) wild type glioblastoma and IDH mutant gliomas	Italy	1, 2	86	NYR	September 2027
Niraparib	NCT06388733	A phase 3, open-label, randomized 2-arm study comparing the clinical efficacy and safety of niraparib with temozolomide in adult participants with newly-diagnosed, MGMT unmethylated glioblastoma	United States	3	450	NYR	March 2028
NMS-03305293 (NMS-293)	NCT04910022	A phase I/II combination study of NMS-03305293 and temozolomide in adult patients with recurrent glioblastoma	United States, Italy, Nether-lands, Switzerland	1, 2	150	R	Nov 2025
Olaparib	NCT03212274	A phase 2 study of the PARP inhibitor olaparib (AZD2281) in IDH1 and IDH2 mutant advanced solid tumors	United States	2	145	ANR	July 2024
Olaparib	NCT02974621	A randomized phase 2 trial of cediranib and olaparib compared to bevacizumab in patients with recurrent glioblastoma who have not received prior VEGF therapy	United States	2	70	ANR	July 2024
Olaparib	NCT03991832	A phase II study of olaparib and durvalumab (MEDI 4736) in patients with IDH-mutated solid tumors	Canada	2	58	R	March 2025
Olaparib	NCT03212742	Phase I/IIa study of concomitant radiotherapy with olaparib and temozolomide in unresectable high grade gliomas patients	France	1, 2	91	R	March 2025
Olaparib	NCT05463848	A surgical “window-of-opportunity” and phase II trial of pembrolizumab, olaparib and temozolomide in recurrent glioblastoma	United States	2	78	R	December 2025
Pamiparib olaparib	NCT04614909	A phase 0/2 clinical trial of pamiparib in newly-diagnosed and recurrent glioblastoma patients	United States	1	30	R	December 2024
Pamiparib (BGB-290)	NCT03914742	Phase I/II study of BGB-290 with temozolomide in recurrent gliomas with IDH1/2 mutations	United States	1, 2	60	C	October 2023
Pamiparib (BGB-290)	NCT03749187	A target validation/phase1 study of BGB-290 in combination with temozolomide in adolescent and young adult IDH1/2 newly diagnosed and recurrent mutant gliomas	United States	1	78	R	July 2029
Talazoparib	NCT04740190	Combination talazoparib - carboplatin for recurrent high-grade glioma with DNA damage repair deficiency (DDRd)	China	2	33	U	December 2023
Veliparib	NCT03581292	A phase 2 study of veliparib (ABT-888) and local irradiation, followed by maintenance veliparib and temozolomide, in patients with newly diagnosed high-grade glioma (HGG) without H3 K27M or BRAFV600 mutations	United States, Australia, Canada, New Zealand, Puerto Rico	2	38	ANR	September 2024
Veliparib	NCT02152982	A phase II/III randomized trial of veliparib or placebo in combination with adjuvant temozolomide in newly diagnosed glioblastoma with MGMT promoter hypermethylation	United States, Puerto Rico	2, 3	447	ANR	December 2024

NYR, not yet recruiting; R, recruiting; ANR, active not recruiting; C, completed; U, unknown.

## Mode of action of PARP inhibitors

In recent years, a lot of research has been dedicated to PARP inhibitors (PARPi), which target poly (ADP-ribose) polymerase 1 (PARP1). PARP1 catalyzes the synthesis of poly (ADP-ribose) (PAR) chains using nicotinamide adenine dinucleotide (NAD^+^) as a substrate. PAR polymers attach to the PARP1 enzyme itself or other proteins that it physically interacts with, including histones and DNA repair factors. PARP1 binds to and gets activated primarily by ssDNA breaks and ssDNA gaps. PAR chains recruit the XRCC1 protein, a scaffold for DNA ligase 3 (LIG3), DNA polymerase β, and polynucleotide kinase 3’-phosphatase (PNKP), which repair the ssDNA breaks. In addition, PARP1 stimulates the repair of dsDNA breaks by recruiting MRE11, a nuclease responsible for end-resection in the homologous recombination (HR) repair pathway. PARP1 can also facilitate dsDNA break repair by non-homologous end joining (NHEJ) and microhomology-mediated end joining (MMEJ). During DNA replication, PARP1 binds to replication forks and can promote fork reversal in response to replication stress. It can also affect chromatin structure to facilitate DNA repair (Ray Chaudhuri and Nussenzweig, 2017).

Although all clinical PARPi bind to the PARP1 catalytic center to prevent NAD^+^ binding and PARylation, they differ in their effect on the adjacent allosteric regulatory domain and in the extent of catalytic inhibition. PARP1 auto-PARylation is required for its rapid release from a ssDNA break. Therefore, strong inhibitors, such as olaparib and talazoparib, tend to “trap” PARP1 on chromatin, resulting in a cytotoxic lesion, although they do not have an allosteric effect on PARP1. Conversely, an allosteric inhibitor veliparib, promotes PARP1 release by inducing changes in PARP1 structure. Although not currently used in clinic, the third type of inhibitors, the “reverse allosteric” inhibitors, trap PARP1 by affecting the inter-domain interactions (Zandarashvili et al., 2020). Overall, PARP1 trapping is associated with a higher cytotoxicity for tumors but also for normal cells (Pommier et al., 2016).

PARP inhibitors were initially discovered to be synthetic lethal in conjunction with HR deficiency, in particular with *BRCA1* and *BRCA2* mutations, which are common in breast and ovarian cancers. However, the identity of the relevant lesions that cause PARPi hypersensitivity of BRCA1/2-deficient cells is the subject of debate. According to the “classical” view, collapsed forks resulting from the absence of the BRCA1/2 fork protective function give rise to dsDNA breaks, which cannot be repaired by BRCA1/2 dependent HR. Since PARP1 plays a major role in ssDNA break repair, PARP1 inhibition leads to an accumulation of ssDNA breaks, which become converted to dsDNA breaks during the second cycle DNA replication (Simoneau et al., 2021), leading to PARP/BRCA synthetic lethality (Bryant et al., 2005; Farmer et al., 2005). Although ssDNA gaps might directly trigger the checkpoint response and block cell division, the ATR checkpoint pathway becomes more dramatically activated in the second S phase following PARPi treatment, presumably by the dsDNA breaks. ATR activation leads to the suppression of replication origin firing and G2/M arrest in the second cell cycle. Notably, BRCA1/2-deficient cells are unable to activate ATR in the second replication cycle (Simoneau et al., 2021). However, a recent report suggests that the critical defect resulting in PARPi hypersensitivity is the inability of BRCA1/2-deficient cells to suppress replication gaps (Cong et al., 2021; Cong and Cantor, 2022). In this scenario, ssDNA gaps rather than dsDNA breaks are the lethal lesion, and dsDNA break appearance might be indicative of the onset of apoptosis instead of being the primary cause of cell death (Cong et al., 2021; Panzarino et al., 2021). The major source of ssDNA gaps in unperturbed cells are Okazaki fragments. PARP1 is involved in the repair of unligated Okazaki fragments (Hanzlikova et al., 2018), and PARPi treatment results in the accumulation of nascent strand discontinuities (Vaitsiankova et al., 2022).

ssDNA gaps can be converted into dsDNA breaks via several mechanisms. For example, ssDNA might fold into secondary structures, such as hairpins, which are amenable to cleavage by nucleases creating fragile sites (Polleys et al., 2023; Whalen and Cantor, 2023). Extensive ssDNA regions can expose distant inverted repeats and lead to gross chromosomal rearrangements (Ait Saada et al., 2023). Post-replicative ssDNA gaps induced by PARPi and BRCA1/2 deficiency are filled by DNA polymerase Θ (POLQ) (Belan et al., 2022; Mann et al., 2022; Schrempf et al., 2022), and POLQ inhibitors act synergistically with PARPi in BRCA1/2 deficient cells (Zatreanu et al., 2021). However, translesion synthesis (TLS) polymerases can also suppress the gaps (Nayak et al., 2020). The relative contribution of different TLS polymerases vs. POLQ to sealing PARPi-induced gaps remains to be determined. Another recently proposed model suggests that PARPi sensitivity of BRCA2-deficient cells is mediated primarily by transcription/replication conflicts (TRCs) and R-loops. According to this model, PARP1 senses TRCs and pauses the replication fork until TRCs are resolved (Petropoulos et al., 2024). Interestingly, PARP1 trapping is not required for TRC induction and trapping reduces PARPi selectivity in killing BRCA-deficient but not normal cells.

Mutated versions of isocitrate dehydrogenase enzymes, cytoplasmic IDH1 and mitochondrial IDH2, are frequently found in gliomas. The mutant enzymes acquire a novel activity that reduces alpha-ketoglutarate (alpha-KG) to 2-hydroxyglutarate (2HG), which inhibits alpha-KG-dependent dioxygenases. These include lysine demethylases (KDMs), which are responsible for demethylating histone tails and chromatin remodeling. Similar to BRCA1/2 deficient cells, IDH1/2 mutant cells are sensitive to PARPi (Sulkowski et al., 2017; Sulkowski et al., 2020). However, unlike BRCA1/2 deficiency, IDH1/2 mutations do not result in genomic instability and accumulation of large deletions or insertions, suggesting that the HR pathway is functional. Instead, IDH1/2 mutants are characterized by increased heterochromatin leading to slowdown of replication forks, replication stress, and the accumulation of ssDNA gaps (Schvartzman et al., 2023). Thus, ssDNA gaps might be the underlying cause of PARPi sensitivity in both IDH1/2 and BRCA1/2 mutants. Several clinical trials are currently ongoing to test PARPi against IDH1/2 mutant gliomas, either as a monotherapy or in combination with TMZ (Fanucci et al., 2023; Sharma et al., 2023) (Table 1).

In conclusion, recent studies highlighted the importance of ssDNA gaps as the primary cytotoxic lesions induced by both BRCA deficiencies and the action of PARPi. The observation that ssDNA gaps are also implicated in the cell killing by TMZ strengthens the rationale for TMZ/PARPi combination therapy.

## TMZ-induced cytotoxic lesions and PARPi effects in TMZ-sensitive cells

We will now examine the effect PARPi might have in TMZ-treated cells depending on the cell DNA repair capacity and the primary cytotoxic lesion induced by TMZ. The outcome of PARPi application is expected to differ depending on the status of three major DNA repair pathways, methylguanine-DNA methyltransferase (MGMT), MMR, and base excision repair (BER). MGMT directly repairs *O* ^*6*^-meG lesions, reverting them to G. In MGMT-positive cells, PARP1 binds to and PARylates MGMT, stimulating its enzymatic activity. Thus, PARPi would reduce MGMT activity and sensitize cells to TMZ (Wu et al., 2021; Cropper et al., 2022). However, MGMT is not expressed in many gliomas, rendering these hypersensitive to very low (∼10 μM) TMZ concentrations.

**FIGURE 1:**
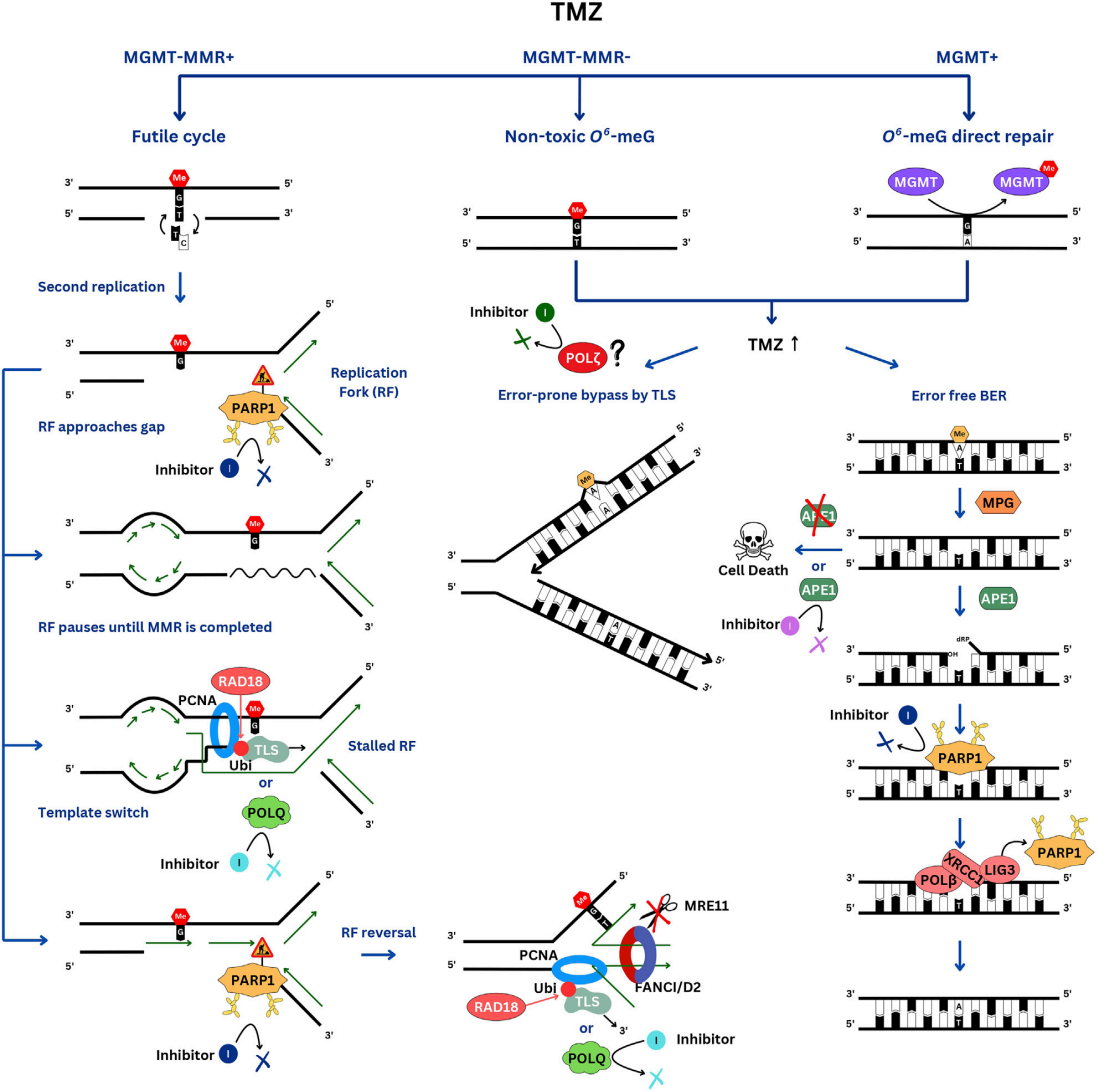
DNA repair pathways involved in TMZ resistance. In the TMZ-sensitive MGMT-negative glioblastomas, the futile cycle of *O* ^*6*^-meG/T repair by MMR results in persistent ssDNA gaps, which interfere with the second S phase. When the replication fork encounters a gap, it pauses. PARP1 is activated by the ssDNA breaks and signals to slow down the fork. If the gap is not filled in a timely manner, the fork will have to be stabilized by reversal and protection from nucleases by the FANCI/D2 DNA clamp. Gaps can be skipped via re-priming and filled after the bulk of DNA replication is completed by the TLS polymerases, which are recruited to RAD18-ubiquitinated PCNA, and by template switching. In MGMT-positive cells, *O* ^*6*^-meG is directly repaired, whereas in MMR-negative cells, replication of *O* ^*6*^-meG/T leads to a C>T substitution without causing a gap. These cells are very resistant to TMZ. At high TMZ concentrations, 3-meA replication blocking lesion accumulates and is repaired by BER or by-passed by TLS. Of note, PARP1 is involved in TMZ resistance in both TMZ-sensitive and resistant cells but via different mechanisms.

In MGMT-negative cells, *O* ^*6*^-meG adducts mispaired with thymine during DNA replication are recognized by the MMR pathway and, via the aforementioned “futile cycle” of MMR give rise to persistent gaps that stall replication forks. Single-stranded DNA breaks, which can be detected in an alkaline comet assay, appear during the first round of DNA replication and persist for many hours afterward. Stretches of ssDNA recruit RPA protein, which in turn binds to ATR kinase, which phosphorylates CHK1, triggering DNA damage checkpoint and cell cycle arrest before the second cell division after the TMZ treatment (Stojic et al., 2004). TMZ sensitivity of MGMT-negative cells appears to primarily depend on their ability to process stalled forks (Figure 1). The most TMZ- (and MNNG-) sensitive among MGMT-negative lines are deficient for Fanconi Anemia (FA) genes (MacLeod et al., 2019; Olivieri et al., 2020; Cheng et al., 2024). The reason for this is not well understood. A probable explanation could be that the FANCD2/FANCI dimer, the key effector of the FA pathway, protects stalled replication forks from nuclease-mediated degradation (Schlacher et al., 2012) and is likely required to rescue the forks colliding with sites of the ongoing *O* ^*6*^-meG-induced futile cycle of mismatch repair. In addition, MMR-induced gaps might persist until mitosis and be resolved in a FANCD2/FANCI-dependent manner to allow for ordered chromosomal segregation. In mitosis FANCD2 specifically localizes to the sites where sister chromatid bridging occurs (Chan et al., 2009). FANCD2, in association with endonucleases (from prophase to metaphase) (Naim et al., 2013) and Bloom syndrome helicase (at anaphase) (Naim and Rosselli, 2009), resolves intermediates resulting from incomplete replication. Knockout of the PCNA ubiquitinating E3 enzyme RAD18 also significantly sensitizes MGMT-negative cells to TMZ, suggesting the involvement of TLS. TMZ induces PCNA mono-ubiquitination in MGMT-negative cells peaking at the time of the second round of DNA replication (Cheng et al., 2024). Based on a CRISPR screen in a RAD18 knockout line, the NHEJ pathway becomes more important for cell survival after TMZ treatment compared to wild type, indicating that ssDNA gaps are converted into dsDNA breaks when not processed by TLS. Interestingly, in combination with TMZ treatment, RAD18 deficiency increases the C>T substitutions resulting from *O* ^*6*^-meG mispairing with thymine but abolishes the TMZ-mediated induction of all other types of single nucleotide variants (SNVs). These results suggest that translesion synthesis plays a role in filling in the ssDNA gaps generated by MMR (Cheng et al., 2024).

Considering the crucial role of ssDNA breaks and gaps in TMZ cytotoxicity, PARP1 might be expected to be involved in their repair through its canonical function in the ssDNA break repair pathway. In addition, PARP1 is involved in the rescue of stalled forks. PARP1 is recruited to replication forks, restrains fork progression under replication stress and facilitates the recruitment of DNA translocases (HLTF, SHPRH, ZRANB3 and SMARCAL1) to reverse damaged forks (Liao et al., 2018; Ho et al., 2022). A reversal of the fork encountering a persistent gap caused by the ongoing futile MMR, might facilitate its bypass. However, PARP1 recruits MRE11 endonuclease (Bryant et al., 2009), which excessively degrades stalled forks in BRCA-mutant cells that are deficient in fork protection. Loss of PARP1 was shown to protect forks from MRE11-mediated degradation and promote cell survival in BRCA1- (Ray Chaudhuri et al., 2016) and BRCA2- (Ding et al., 2016) deficient cells. Thus, based on its various roles in replication fork remodeling, PARP1 might either promote cell survival or exacerbate TMZ toxicity.

There is limited evidence that PARP1 might play a role in the MMR pathway (Liu et al., 2011; Paul et al., 2023). Hypothetically, PARPi might inhibit MMR at an intermediate step, e.g., ssDNA gap, potentially enhancing TMZ sensitivity. In addition, MMR and PARP1 interact at G-quadruplexes (Cong et al., 2024; Isik et al., 2024). The question of PARP1 involvement in MMR remains underexplored and deserves further investigation.

Surprisingly, in the same CRISPR-based screens that revealed the critical role of the FA genes in TMZ/MNNG resistance, PARP1 depletion did not hyper-sensitize MGMT-negative glioblastomas to TMZ (MacLeod et al., 2019) and led to only a minor increase in MNNG sensitivity in RPE1 cells (Olivieri et al., 2020). Nevertheless, the non-trapping PARPi veliparib potentiated TMZ toxicity in MGMT-negative gliomas *in vitro* and in a mouse xenograft model (Gupta et al., 2014). The question of whether PARP inhibitors indeed increase TMZ sensitivity in MGMT-negative cells treated with low TMZ concentrations by inhibiting PARP1 [and not, e.g., other PARP proteins as was suggested in one recent study (Higuchi et al., 2020)] deserves careful investigation using *PARP1* isogenic knockouts. In addition, it remains to be determined if PARP1 trapping is relevant to the action of PARPi in MGMT-negative cells treated with TMZ.

## TMZ and PARPi effects in TMZ-resistant cells

TMZ-treated glioblastomas acquire resistance and recur in almost 100% of cases. MMR inactivation, which abolishes futile cycles of *O* ^*6*^-meG/T repair and the associated ssDNA gaps, leads to an extraordinary TMZ tolerance (1 mM TMZ and higher). A hypermutator phenotype associated with MMR deficiency was reported for recurrent gliomas in 60% of TMZ-treated patients (Johnson et al., 2014). Interestingly, TMZ resistance was found to readily build up in cell culture in the MGMT-negative but not in the MGMT-positive glioma lines upon as few as two 3-day cycles of TMZ treatment (Perazzoli et al., 2015). The *in vitro* acquisition of resistance was accompanied by the downregulation of MMR protein expression. However, the upregulation of MGMT in MGMT-negative lines was observed only rarely or not at all (Perazzoli et al., 2015; Yuan et al., 2020). Interestingly, PARPi prevented the emergence of TMZ resistance *in vitro* (Yuan et al., 2020). The rapid acquisition of TMZ resistance concomitant with MMR downregulation hints that an epigenetic mechanism might be responsible.

In MMR-negative cells *O* ^*6*^-meG is rendered non-toxic since, when left unrepaired it doesn’t affect the replication process and only results in C>T substitutions (Figure 1). Instead, other DNA base adducts accumulate in cells, which become exposed to higher TMZ concentrations. In these cells, TMZ toxicity is mediated mainly by a replication-blocking 3-meA, which is either removed by BER or bypassed by TLS. In addition, 7-meG, although non-toxic and non-mutagenic by itself, also triggers BER and might lead to the accumulation of toxic repair intermediates. BER is a preferred repair pathway for cell survival, since it is error-free and, if completed prior to DNA replication, precludes replication problems. During BER, 3-meA base is first excised by the N-methylpurine DNA glycosylase (MPG, also known as alkyl adenine DNA glycosylase, AAG), resulting in an abasic site, which is then cleaved by the apurinic/apyrimidinic site endonuclease (APE1). PARP1 efficiently binds to BER intermediates with a 5’-deoxyribose phosphate (5’-dRP) end, which are generated by the APE1 cleavage (Cistulli et al., 2004). Interestingly, PARP1 and the PAR-binding chromatin remodeler ALC1/CHD1L also promote chromatin accessibility to APE1 (Hewitt et al., 2021; Verma et al., 2021). Methyl methanesulfonate (MMS) and PARPi sensitivity of the *ALC1−/−* cells is partially rescued by inhibiting BER via MPG depletion (Hewitt et al., 2021). Therefore, PARP1 activation might be triggered not only by ssDNA breaks but also by the abasic sites resulting from the excision of the alkylated bases by the MPG. However, PARP1 is not essential for BER (Ray Chaudhuri and Nussenzweig, 2017) and in this context, PARP1 trapping is more detrimental to cell survival than inhibiting its catalytic activity. PARPi olaparib induces more PARP1 trapped on chromatin than veliparib, which makes it more efficient in potentiating TMZ cytotoxicity in TMZ-resistant cells (Murai et al., 2014). XRCC1 plays a dual role of releasing PARP1 from chromatin, thus limiting its activity, and recruiting LIG3 and POLB to complete BER. *XRCC1−/− PARP1−/−* double knockout cell line is more resistant to MMS, which induces mainly 3-meA and 7-meG, than either *PARP1−/−* or *XRCC1−/−* single knockouts. Thus, it appears that promoting the release of PARP1 from the DNA is a more important aspect of XRCC1 function than serving as a scaffold for LIG3 and POLB (Demin et al., 2021). A likely explanation for the toxicity associated with XRCC1 deficiency is excessive PARP1 activity leading to NAD^+^ depletion and cell death by an “energetic catastrophe,” which is prevented in *XRCC1−/− PARP1−/−* double knockout. Compared to TMZ, MMS induces more 3-meA and 7-meG and less *O* ^*6*^-meG, thus mimicking TMZ effects in MGMT-positive or MMR-deficient cells. Remarkably, in the MGMT-positive MCF-7 cell line, *XRCC1−/−*, *PARP1−/−* double knockout is also much more resistant to TMZ than *XRCC1−/−* and *PARP−/−* singles, similar to what was observed with MMS (Hirota et al., 2022). However, in MGMT-negative TK6 cells, the differences in TMZ sensitivities among the three knockouts are very modest (Hirota et al., 2022), consistent with 3-meA playing only a minor role in inducing MGMT-negative MMR-proficient cell lethality compared to *O* ^*6*^-meG.

## MMS, a well-studied model of TMZ treatment of MMR-deficient cells

The response to the experimental methylating agent MMS was the subject of detailed studies in the past. As opposed to TMZ, MMS belongs to the group of S_N_2 (second-order nucleophilic substitution) methylating agents and leads to 3-meA and 7-meG and less *O* ^*6*^-meG. MMS is not used in the contemporary clinical practice. Compared to MMS, TMZ displays superior pharmacokinetic properties, e.g., TMZ is readily absorbed in the intestinal tract, penetrates the blood-brain barrier, and is a prodrug converted into an active intermediate with the clinically suitable kinetics.

Based on early research in yeast, MMS was considered a radiomimetic, inducing dsDNA breaks that are repaired by HR. However, it was later demonstrated that the dsDNA breaks, which had been observed following MMS treatment, were an experimental artefact caused by *in vitro* handling of the DNA samples and the requirement of HR proteins for MMS resistance in budding yeast most likely stems from the necessity to protect the stalled replication forks (Lundin et al., 2005). Although BER is the optimal repair pathway of 3-meA, upon treatment with high concentrations of MMS, it may become overwhelmed by the number of lesions, and replication forks may start encountering unrepaired 3-meA adducts, causing them to stall. In *Xenopus* cell-free extracts, it was found that replication of MMS-damaged DNA results in the accumulation of ssDNA gaps behind the fork, which are then either filled in by TLS or repaired via template switching (TS) in a RAD51-dependent manner (Hashimoto et al., 2010). Without fork-protecting RAD51, MMS-induced ssDNA gaps behind replication forks were greatly enhanced and generated via the MRE11-mediated degradation of the native strand in the stalled forks (Hashimoto et al., 2010). This result suggests that fork protection might play an important role in MMS resistance and, by inference, in resistance of MGMT-positive or MMR-deficient lines to TMZ. However, FA pathway deficiencies do not sensitize MGMT-positive glioblastomas to TMZ (MacLeod et al., 2019). Similarly, RAD18-mediated TLS doesn’t appear to play a role in either MGMT-positive or MMR-deficient glioblastoma lines (Cheng et al., 2024). Most likely, the fork encounter with the MMR-generated gaps necessitates a different kind of response compared to a small replication-blocking adduct, like 3-meA.

Similar to the lack of FA pathway requirement, BRCA1 doesn’t appear to be important for fork protection with regard to resistance to 3-meA. Although *BRCA1* knockout cells display hypersensitivity to MMS, this is reversed by *53BP1* deficiency (Cong et al., 2021). According to the “traditional” point of view, inactivation of *53BP1* would facilitate resection of dsDNA breaks and HR, implicating dsDNA breaks as the MMS-induced lethal lesion. However, an alternative hypothesis postulates that 53BP1 deficiency restores Okazaki fragment processing (OFP), which is defective in *BRCA1* mutants (Cong et al., 2021). In this scenario, MMS induces additional gaps, which synergize with the endogenous ones caused by the OFP defect. MMS also leads to PARP1 activation via BER-induced abasic sites (Hewitt et al., 2021). Importantly, the loss of 53BP1 rescues HR, PARPi hypersensitivity, and embryonic lethality of mice with mutant *BRCA1* alleles but not the defects of interstrand crosslink repair or replication fork stabilization (Bunting et al., 2010; Bunting et al., 2012; Li et al., 2016). It is worth noting that the rescue is not complete since the *53BP1−/−* double knockout mice with null rather than mutated *BRCA1* alleles, while viable, still display an HR defect and PARPi sensitivity (Chen et al., 2020). PARPi resistance of BRCA1/53BP1 double deficient cells can be reversed by depletion of LIG3, which leads to the accumulation of PARPi-induced ssDNA gaps (Paes Dias et al., 2021). Interestingly, ssDNA gaps in PARPi-treated BRCA1-deficient cells appear to be different from the gaps in BRCA1/53BP1 double deficient cells with depleted LIG3, since only the latter but not the former are suppressed by the inhibition of MRE11. This observation highlights the general trend that although all ssDNA gaps might activate PARP1, they differ in their origin and the response they elicit.

In *Xenopus* extracts (Hashimoto et al., 2010), as well as in yeast (Lopes et al., 2006), MMS has little effect on the ssDNA gaps directly at the forks, which result from the uncoupling of the leading and lagging strand synthesis or uncoupling of the replicative helicase and polymerase. It is also worth noting that only very few reversed forks were observed in MMS-treated yeast (Prado, 2018), presumably because 3-meA lesions are easily bypassed, and fork reversal might be very transient. Nevertheless, knockdown of a DNA translocase with fork-reversing activity, ZRANB3, was reported to sensitize human cells to MMS (Weston et al., 2012), suggesting that fork reversal might still contribute to MMS resistance. Overall, it appears that in the case of 3-meA, ssDNA gap repair behind the fork via TLS and TS might be more important for lesion tolerance compared to fork reversal or fork protection. In yeast, TS is regulated by a Rad5, a bi-functional enzyme with PCNA poly-ubiquitin ligase and DNA translocase activities. Two RAD5 orthologues, HLTF and SHPRH, exist in human cells. MMS treatment was reported to result in the degradation of HLTF and an enhanced SHPRH association with Rad18 and TLS polymerase κ, which is capable of more error-free bypass of MMS-induced lesions than a competing polymerase η (Lin et al., 2011). However, in a more recent study, knockout of HLTF resulted in an increased MMS mutagenesis, while knockout of SHPRH did not change the mutation frequency and increased MMS resistance (Seelinger et al., 2020). Since both studies determined the rate of MMS-induced mutations using MMS-treated plasmids transfected into human cells, the number of analyzed mutations was generally small. With the advent of an affordable whole genome sequencing (WGS), it will be important to re-investigate the error rates of different TLS polymerases bypassing MMS- and TMZ-induced DNA base adducts *in vivo* and their contribution to TMZ resistance. Interestingly, a recent report uncovered that upregulation of TLS by DNA polymerase ζ suppresses MMS hypersensitivity in yeast deficient for a replisome component Ctf4, which is normally involved in TS (Dolce et al., 2022).

## Inhibition of BER as a TMZ-potentiating strategy

If replication is initiated before BER repair of methylated bases is complete, replication fork-BER conflicts could ensue. It is presumed that an encounter of the replication fork with the BER-induced ssDNA gaps will result in fork collapse and dsDNA breaks, although, to our knowledge, this issue has yet to be investigated. In order to differentiate the direct effects of 3-meA from BER-induced gaps, it will be important to perform experiments with a set of triple knockouts of DNA repair genes in *MSH2−/− MPG−/−* genetic background. The use of these cell lines in combination with WGS will allow evaluating the role of TLS and TS in TMZ resistance and mutagenesis. Similar experiments performed with a MMS-treated set of yeast double deletion mutants with *mag1* (yeast orthologue of *MPG*) more than a decade ago, suggested that the yeast translocase Rad5, TLS polymerases η and ζ, as well as human polymerases κ, and ι, when expressed in yeast, can all mediate replication through 3-meA (Johnson et al., 2007).

Overall, interfering with the BER pathway appears to be the most efficient route to re-sensitize MMR-deficient cells to TMZ and thus prevent the build-up of TMZ resistance through MMR inactivation. In MMR-deficient cells treated with high TMZ concentrations, the effect of PARPi would be primarily to impede the completion of BER by blocking access to the transient ssDNA breaks, which are formed in the process, and the potential role of PARPi in the inhibition of replication fork rescue is likely to be of secondary importance. PARP inhibition leads to synergistic cytotoxicity with the TMZ treatment in cultured MMR-deficient cells, which is even more pronounced than in MMR-proficient cells (Gupta et al., 2014). However, *in vivo*, the PARPi/TMZ combination therapy faces problems with not achieving an effective concentration of PARPi in the brain (Gupta et al., 2014) and due to hematological toxicity, which ensues when the drugs are used together (Gueble et al., 2022). A new generation of PARPi with improved blood-brain barrier penetrance, high PARP1 inhibition potency, enhanced PARP1 vs. PARP2 selectivity, and reduced toxicity to normal cells are currently being tested, and might overcome this limitation (Gueble et al., 2022).

Since BER is activated in response to 3-meA and 7-meG, blocking it at an intermediate step would result in toxic intermediates, such as abasic sites or ssDNA breaks. The second step of BER, the conversion of the abasic site into ssDNA break, which is catalyzed by the APE1 endonuclease, appears to represent an Achilles’ heel of this repair pathway. MMS treatment of yeast deficient in APE1 orthologues, *APN1* and *APN2*, leads to dsDNA breaks even without DNA replication (Ma et al., 2011). A combination of TMZ with an APE1 inhibitor should result in the accumulation of 3-meA and 7-meG-induced abasic sites in tumor clones that managed to overcome *O* ^*6*^-meG toxicity via MMR inactivation. Elimination of these clones would prevent the build-up of TMZ resistance. Indeed, in an exceptional case, TMZ treatment cured a patient of glioblastoma, carrying an *APE1* gene rendered inactive by a translocation (Wheeler et al., 2021). It is worth noting that APE1 inhibition would be equally expected to sensitize MGMT-positive gliomas to TMZ.

Several substances were reported to act as APE1 inhibitors. Methoxyamine forms a stable adduct with an abasic site and renders it refractory to cleavage by APE1. It enhances TMZ cytotoxicity in MGMT-positive but not in MGMT-negative glioblastomas *in vitro*, but the required concentration (20 mM) is very high (Montaldi and Sakamoto-Hojo, 2013), and clinical studies were unsuccessful (Malfatti et al., 2023). Lucanthone was initially developed as an anti-schistosome agent and a topoisomerase II inhibitor but was later shown to inhibit recombinant APE1 protein *in vitro* (Luo and Kelley, 2004; Naidu et al., 2011) and at micromolar concentrations sensitizes cultured cells to TMZ (Luo and Kelley, 2004). Lucanthone is able to cross the blood-brain barrier and has been tested as an adjuvant for brain tumor radiotherapy (Del Rowe et al., 1999). However, it likely has several targets besides APE1 and was reported to inhibit autophagy in gliomas (Radin et al., 2022; Radin et al., 2024). Thus, the mechanism of lucanthone action remains uncertain.

High-throughput screens for the inhibitors of the APE1 abasic site endonuclease activity identified several compounds [reviewed in Malfatti et al. (2023)]. Two of them (Madhusudan et al., 2005; Rai et al., 2012) became commercially available, however, recent studies cast doubt on their specificity and efficacy (Xue and Demple, 2022; Pidugu et al., 2023). As of today, none of the compounds, which were isolated in the screens, advanced to clinical trials (Malfatti et al., 2023). The most recent effort using high-throughput X-ray crystallography screen of the fragment library binding to APE1 crystals resulted in the development of two lead compounds targeting the APE1 endonuclease domain with IC_50_ values below the micromolar range (Das et al., 2023). This new approach might facilitate the development of novel APE1 inhibitors to be tested in clinical trials.

## Concluding remarks

As of today, our knowledge of TMZ response is mostly limited to BER in TMZ-resistant MMR-deficient or MGMT-positive cells. The mechanism of cell death triggered by “futile MMR cycles” in primary TMZ-sensitive glioblastomas remains largely enigmatic. While it appears that ssDNA gaps are generated and TLS is involved in both TMZ-sensitive and TMZ-resistant cells, the identity of a TLS polymerase is likely to be different since RAD18-dependent PCNA ubiquitination plays an important role in the former but not in the latter (Cheng et al., 2024). It is possible but remains to be tested if MMR-generated gaps are filled in by a Y-family polymerase (η, κ or ι) while 3-meA-induced gaps are dealt with by the polymerase ζ, which can be recruited to chromatin in a ubi-PCNA-independent manner via REV1. A recently developed REV1 inhibitor, JH-RE-06, targets REV1/polymerase zeta complex formation (Wojtaszek et al., 2019). Given that JH-RE-06 synergizes with PARPi to kill BRCA-deficient cells (Taglialatela et al., 2021), it might be worth testing in combination with TMZ. Overall, finding new approaches to re-sensitize TMZ-resistant cells might be a better strategy than to hyper-sensitize primary TMZ-sensitive glioblastomas, which already withstand only very low TMZ concentrations. If inactivation of MMR doesn’t increase the odds of tumor survival in combination therapy, because cells are rendered hypersensitive to 3-meA by the second drug, the problem of TMZ resistance may be solved. In this regard, targeting APE1 holds a great promise. Alternatively, since WRN helicase plays an essential role in MMR-deficient cells, treatment with a WRN inhibitor (Baltgalvis et al., 2024) might eliminate the emerging TMZ-resistant MMR-deficient glioblastoma cells.

There are certain parallels between BRCA deficiencies and TMZ treatment. Both are currently believed to induce ssDNA gaps, which underlie sensitivity to PARPi. While the origin of the gaps is obviously different, certain features of the cellular response to the gaps might be universal, such as PARP1 signaling or post-replicative fill-in by TLS or POLQ. Thus, TLS and POLQ inhibitors, which were reported to synergize with PARPi in targeting BRCA-deficient cells (Taglialatela et al., 2021; Zatreanu et al., 2021), might potentiate TMZ as well, especially as a three-drug combination with PARPi. Future studies will shed light on the feasibility of this approach.
